# BM-MSC Transplantation Alleviates Intracerebral Hemorrhage-Induced Brain Injury, Promotes Astrocytes Vimentin Expression, and Enhances Astrocytes Antioxidation via the Cx43/Nrf2/HO-1 Axis

**DOI:** 10.3389/fcell.2020.00302

**Published:** 2020-05-08

**Authors:** Xiao Chen, Huaibin Liang, Zhiyu Xi, Yong Yang, Huimin Shan, Baofeng Wang, Zhihong Zhong, Canxin Xu, Guo-Yuan Yang, Qingfang Sun, Yuhao Sun, Liuguan Bian

**Affiliations:** ^1^Department of Neurosurgery, Ruijin Hospital, School of Medicine, Shanghai Jiao Tong University, Shanghai, China; ^2^Department of Neurology, Shanghai Ninth People’s Hospital, School of Medicine, Shanghai Jiao Tong University, Shanghai, China; ^3^Department of Neurosurgery, Guangdong General Hospital, Guangdong Academy of Medical Sciences, Guangzhou, China; ^4^Neuroscience and Neuroengineering Center, Med-X Research Institute and School of Biomedical Engineering, Shanghai Jiao Tong University, Shanghai, China

**Keywords:** ICH, MSC, astrocytes, Cx43, Nrf2

## Abstract

Intracerebral hemorrhage (ICH) is a particularly severe form of stroke, and reactive astrogliosis is a common response following injury to the central nervous system (CNS). Mesenchymal stem cells (MSCs) are reported to promote neurogenesis and alleviate the late side effects in injured brain regions. Gap junctions (Gjs) are abundant in the brain, where the richest connexin (Cx) is Cx43, most prominently expressed in astrocytes. Nuclear factor erythroid 2-related factor 2 (Nrf2) is an essential transcription factor regulating antioxidant reactions. Here, we aimed to explore whether bone marrow MSCs (BM-MSCs) could alleviate brain injury and protect astrocytes from apoptosis, by regulating Cx43 and Nrf2. We validated the effect of BM-MSC transplantation in an ICH model *in vivo* and *in vitro* and detected changes using immunofluorescence, as well as protein and mRNA expression of glial fibrillary acidic protein (GFAP), vimentin (VIM), Cx43, Nrf2, and heme oxygenase-1 (HO-1). Our results showed that BM-MSC transplantation attenuated brain injury after ICH and upregulated VIM expression *in vivo* and *in vitro*. Additionally, Cx43 upregulation and Nrf2 nuclear translocation were observed in astrocytes cocultured with BM-MSC. Knockdown of Cx43 by siRNA restrained Nrf2 nuclear translocation. Cx43 and Nrf2 had a connection as determined by immunofluorescence and coimmunoprecipitation. We demonstrated that astrocytes undergo astroglial-mesenchymal phenotype switching and have anti-apoptotic abilities after BM-MSC transplantation, where Cx43 upregulation triggers Nrf2 nuclear translocation and promotes its phase II enzyme expression. The Cx43/Nrf2 interaction of astrocytes after BM-MSC transplantation may provide an important therapeutic target in the management of ICH.

## Introduction

Intracerebral hemorrhage (ICH) is a severe form of stroke with high mortality and morbidity, and survivors usually have profound neurological deficits (Qureshi et al., 2009; Keep et al., 2012). It is the second most common form of stroke, accounting for about 10–30% of cases, and the major causes of disability and death (Bedini et al., 2018). To date, despite the considerable scientific progress achieved by animal and preclinical studies, there remains no effective therapeutic strategy for ICH patients, except for active rehabilitation (Broderick et al., 1994). Two different processes describe the cascade of injuries caused by ICH. In the primary injury, mechanical compression results in the destruction of neurons and glial structures, resulting in impaired neurotransmitter release, mitochondrial dysfunction, and ultimately apoptosis, edema, inflammation, and necrosis (Graham et al., 2000; Qureshi et al., 2003). The secondary injury is characterized by hemoglobin breakdown products activating hemostatic cascades, and presence of clots is often accompanied by the activation of astrocytes, resulting in dense glial scars, which exacerbate neurological deterioration and affect long-term neuronal recovery (Aronowski and Zhao, 2011; Zhou et al., 2014; Moeendarbary et al., 2017).

Astrocytes are the most important glial cells in the central nervous system (CNS), providing nutrition and maintaining structure for neurons, which also contributing to keeping homeostasis of the extracellular environment (Sofroniew and Vinters, 2010; Macco et al., 2013; Yang et al., 2018). When the CNS is damaged due to nerve injury, infection, ischemia, hemorrhagic stroke, or neurodegeneration, astrocytes are activated, prior to proliferating, migrating, and finally forming glial scars (Anderson et al., 2014). Further study on the potential mechanisms of astrocyte activation could help to improve the prognosis of patients with CNS disorders.

Mesenchymal stem cells (MSCs) are the prototype of pluripotent stem cells, and have potential applications in regenerative medicine. MSCs are self-renewing and multipotent cells capable of generating progenitors and differentiating into various and distinct cell lineages, which can be isolated from different human tissues, amplified and/or differentiated *in vitro*, and then treated and administered as stem cell-based drugs to patients. The repair mechanisms of MSCs are mainly related to their nutritional effects on other cells, as well as their highly anti-inflammatory and immunomodulatory capabilities (Liao et al., 2009; Uccelli et al., 2011; Zhang et al., 2013). Previous studies have also shown that MSCs have antioxidative stress properties (Shalaby et al., 2014). Several studies have revealed that bone marrow MSCs (BM-MSCs) can interact with the injured microenvironment and shift the balance from toxic to protective regenerative events through the release of bioactive factors (Liu et al., 2018). These special abilities make them a very potent potential treatment for several CNS diseases, such as ischemic stroke (Zhao et al., 2002) and ICH (Seyfried et al., 2006). Ongoing research efforts are increasing our understanding of the cellular and molecular mechanisms by which MSCs could promote neurogenesis and alleviate late side effects in injured brain regions, including through strengthening of the vascular system, and restoring motor, sensory and cognitive functions (Bedini et al., 2018).

The nuclear factor erythroid 2-related factor 2 (Nrf2) antioxidant response element (ARE) signaling pathway is one of the most important cellular defense mechanisms against oxidative stress, regulating the antioxidant reaction caused by reactive oxygen species (ROS) (Itoh et al., 1999; Shih et al., 2003). After encountering oxidative and electrophilic stimulation, Nrf2 would transfers from the cytosol into the nucleus, then combines with and activates antioxidant response (ARE), which produces a protective cell response characterized by high expression of antioxidant enzymes such as NAD(P)H quinone oxidoreductase 1 (NQO1), Heme oxygenase-1 (HO-1) and Superoxide Dismutase 2 (SOD2) (Deng et al., 2015; Liu et al., 2015; Kleszczynski et al., 2016). HO-1 has been shown to have a cytoprotective effect on oxidative and inflammatory stress, with an important role in metabolic function and cell protection (Parada et al., 2014; Liu et al., 2015). It also has been reported that MSCs could be used as an antioxidant, via the Nrf2 signaling pathway, to remove ROS and other harmful substances and protect the body from oxidative stress in many diseases, such as acute lung injury, acute respiratory distress syndrome, myocardial infarction, and acute liver failure (Mahrouf-Yorgov et al., 2017; Zhang et al., 2017; Yan et al., 2019).

Gap junctions (Gjs) are abundant in the brain, where the richest connexin (Cx) is Cx43, which is most prominently expressed in astrocytes. Every set of six Cx monomers form a connexon or hemichannel in the plasma membrane, and the interaction between two opposing hemichannels results in gap junction channels. The hemichannels are usually closed, but may open with cell depolarization or with changes in intracellular and extracellular calcium concentration changes, phosphorylation, or redox status changes (Schulz et al., 2015). Once the two closed hemichannels open, direct access to the channels of two adjacent cytoplasms allows the exchange of ions and small molecules, which facilitates the transmission of electrical signals between neurons, as well as chemical signals and metabolites between astrocytes. This syncytial structure of astrocytes, based on Cx43, is essential for maintaining cellular stability. Gjs can also protect healthy neighboring cells from damage or death by providing essential nutrients and metabolites and limiting the spreading of injury (Garcia-Dorado et al., 2004; Decrock et al., 2009). In physiological and pathological states, the function of Cxs is not associated solely with their roles as channels, but also with their role in intracellular signaling (Le et al., 2014; Chen et al., 2017; Yang et al., 2018). A recent study has reported that a lack of Cx43 expression could lead to increased astrocyte death. Le et al. (2014) also showed that Gjs mediated by Cx43 in astrocytes has a positive effect on resistance to oxidative stress. Chen et al. (2017) have also revealed that Cx43 could regulate Nrf2/ARE signaling to resist oxidative stress injury in glomerular mesangial cells.

The aim of this study was to explore whether BM-MSCs can alleviate brain injury and protect astrocytes from apoptosis by regulating Cx43 and Nrf2. We used both *in vivo* and *in vitro* methods to test our hypothesis.

## Materials and Methods

### Experimental Design

The animal experiment protocol was approved by the Animal Care and Use Committee of Ruijin Hospital, Shanghai Jiao Tong University. Animals were maintained in separate cages at room temperature with free access to food and water under a 12/12 h light/dark cycle. Adult male C57BL/6 mice aged 6–8 weeks, weighing 22–25 g were random divided into three groups: (1) group 1, sham (*n* = 48), (2) group 2, ICH + PBS treated (*n* = 55), and (3) group 3, ICH + BM-MSCs treated (*n* = 50) group. At 1, 3, 7, 14 days following BM-MSCs transplantation, neurological score and behavioral experiments were carried out before mice were sacrificed. Brain samples were collected for further experiments. The experimental schematic diagram *in vivo* is shown in Figure 2A.

The *in vitro* experiment was performed as follows: primary astrocytes were seeded on 6/12/24/96-well plates, with or without BM-MSCs coculture via a transwell system (3.0 μm Pore Size, Corning, United States), then exposure to 30 μM hemin for 24 h, as shown in Figure 4A. The physiological changes of astrocytes were analyzed according to the proportion of BM-MSCs coculture (0, 1:1000, 1:100, 10) and time of BM-MSCs coculture (0, 12, 24, 48 h).

### Isolation and Identification of BM-MSCs

BM-MSCs got from Sprague Dawley (SD) rats (Jiesijie, Co., Shanghai, China) weighing 200–230 g as previously described (Horwitz et al., 2002). The femurs and tibias were separated, removing the attached muscles and ligaments as much as possible, then washed thoroughly with phosphate-buffered saline (PBS) to remove blood cells. Marrow cavities of tibias and femurs were flushed with PBS to harvest BM-MSCs (Nagaya et al., 2005). The cells were cultured by Dulbecco’s modified Eagle’s medium (DMEM; Gibco Laboratories, Grand Island, NY, United States) with 10% fetal bovine serum (FBS; Gibco) and 100 U/mL penicillin/streptomycin (Gibco), kept at 37°C with 95% humidity and 5% carbon dioxide. Non-adherent cells were removed after 48 h and then fresh medium was added. The primary BM-MSCs (passage 0) get confluence at 6–7 days, then passaged at a ratio of 1: 2. Pure passage 2–5 was used for the following experiments.

Flow cytometric analysis of cell surface markers was applied for identification as described previously. BM-MSCs were trypsinized into single-cell suspension and stained with first antibodies including anti-rat CD34, CD45, CD29, and CD90 (Cyagen Biosciences Inc., Guangzhou, China) for 30 min, then incubated with the secondary goat anti-mouse-FITC antibody for another 30 min (Cyagen). After washed with PBS three times, the cells were suspended in 300 μl PBS for identification by flow cytometry (BD Biosciences, Mississauga, ON, Canada).

### ICH Models, BM-MSCs Labeling and BM-MSCs Transplantation

**FIGURE 1:**
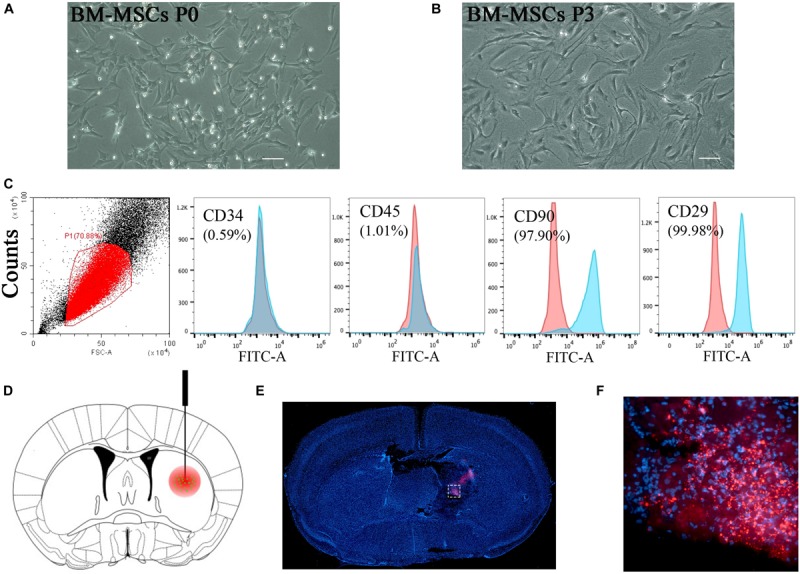
BM-MSCs isolation, identification, and transplantation. **(A,B)** Morphology of MSCs in cell culture. Cultured cells (passage 0 and passage 3) showed typically spindle-shaped morphology under phase-contrast microscopy. Bar = 50 μm. **(C)** Flow cytometry analysis of BM-MSCs at passage 3 depicted that cultured cells were negative for CD34 and CD45, and positive for CD90 and CD29. **(D)** Schematic diagram of BM-MSCs (green) stereotactically injected into the lesion area (red). **(E)** Red fluorescent (PKH26 dye) cells were located in the lesion area after 3 days of injection. Bar = 100 μm. **(F)** Magnification pictures of the injured side.

ICH was successfully produced by the subjection of collagenase IV (Sigma-Aldrich) with 0.075 U dissolved in 0.4 μL PBS as early described (Yang et al., 2018), briefly, 0.5 mm anterior, 3.5 mm ventral, and 2.2 mm lateral to the bregma with a rate of 0.1 μl/min, as shown in Figure 1D. After placement for another 5 min, the needle was gently removed to avoid reflux. The burr hole was sealed by bone wax, and the wound was sutured. Sham-operated mice underwent the same procedures without the injection of collagenase IV. All animals recovered on a heating pad at 37°C after the operation until consciousness back.

PKH26 red fluorescent cell linker mini kit (PKH26, Sigma) was applied for tracking after transplantation. BM-MSCs were trypsinized and resuspended with 2 μM PKH26 dye at room temperature (RT) for 5 min according to the manufacturer’s instructions. We divided the mice with collagenase injection into two groups at 24 h after ICH. For the ICH + MSCs treated group, 2 × 10^6^ BM-MSCs in 2 μl PBS were stereotactically injected into the ipsilateral lesion area with a rate of 0.1 μl/min and placed for another 5 min thereafter. For the ICH + PBS treated group, an equal volume of PBS was administrated to the same position.

After deeply anesthetized, mice were perfused with saline followed by fixation with 4% paraformaldehyde (PFA) at 1, 3, 7, and 14 days after BM-MSCs transplantation. Brains were stored in −80°C refrigerators. Brain cryosections (20 μm) were prepared and subjected to immunofluorescence staining. At the same time points, the peri-lesion area of the mice was applied for Western blotting (WB) analysis and qRT-PCR.

### Isolation and Culture of Primary Cortical Neurons

Primary cortical neurons were obtained from fetal C57BL/6 mice (embryonic day 16 – 18). After removing meninges and blood vessels as much as possible, the remaining cortical tissues were gently ground with 0.25% trypsin and digested at 37°C for 10 min, then plated on transwell chambers at a density of 70,000 cells/cm^2^. Neuronal cells were cultured using Neurobasal medium, supplemented with 1% B27 and 100 units/mL penicillin/streptomycin, and kept at 37°C at 95% humidity and 5% carbon dioxide (CO2). Experiments were performed at 10 – 12 days after plating.

### Isolation and Culture of Primary Astrocytes, and Coculture With BM-MSCs or Neurons

The primary astrocytes were prepared from the pallium of newborn C57BL/6 mice, within 24 h after birth (Jiesijie). The isolation and culture of primary astrocytes performed as previously described (Yang et al., 2018). In brief, after removing meninges and blood vessels as much as possible, the remaining cortical tissues were gently ground with 0.25% trypsin and digested at 37°C for 10 min, then plated on a 75cm^2^ flask coated with poly-D-lysine (Corning, United States) at a density of 20,000 cells/cm^2^, and kept at 37°C at 95% humidity and 5% carbon dioxide (CO_2_). The cells fused in 13–14 days, and half of the media was replaced by fresh media every 4 days. Pure passage 2–5 was used for the following experiments. Astrocytes were incubated into 6/12/24/96-well plates and cocultured with BM-MSCs or Neurons in the transwells system. After 24 h, the cells were exposed to 30 μM hemin to imitating ICH *in vitro* for further experiments and detections.

### Brain Water Content

At 3 days MSCs transplantation, mice were deeply anesthetized, then the entire brain tissues were removed at once. Then the samples were divided into the following five groups: contralateral cortex (Con-Cx), contralateral basal ganglia (Con-BG), ipsilateral cortex (Ipsi-CX), ipsilateral basal ganglia (Ipsi-BG) and cerebellum. Brain tissues were immediately separated and weighed, and the wet weight was recorded. The above five parts were then dried for 72 h at 100–105°C until the tissue water vapored completely to obtain the dry weight. The percentage of water content = (wet weight - dry weight)/wet weight) ^∗^ 100%.

### Brain Hematoma Volume

After mice deeply anesthetized, brains were removed immediately, put in −20°C for 1 h, and then cut into five sections. Brain slices were mounted on dry paper and photographed. Hematoma volume was the sum of all lesion areas multiplied by slice thickness using ImageJ 1.6.0 (NIH, United States) (Wei et al., 2017).

### Behavioral Assessments

Behavioral assessments were determined by the rotarod test and the modified Neurological Severity Scores (mNSS), and performed at 3, 7, 14 days after BM-MSCs transplantation, carried out by a partner unaware of the treatment conditions. The rotarod test was performed using an accelerating rotarod (Bio-will Co., Ltd., Shanghai, China). In brief, mice were placed on the rotarod cylinder, and the time that the mice remained on the rotarod was measured. The speed of rotation (rotation/minute) increases slowly from 4 to 40 in 5 min. Four days before ICH, the mice were trained for three trials/day for 4 days, each trial lasted for 5min with a 2 h interval between each trial, and the maximum time score was recorded 1 day before ICH induction. The mNSS ranging from 0 to 14 score, consists of beam balance tests (0 – 6), walking on the floor (0 – 3), raising mice by the tails (0 – 3), and response absence (0 – 2). According to the scoring criteria (Cheng et al., 2018), the higher the score, the more serious the injury.

### Drug Administration and siRNA Transfection

We dissolve hemin (Aladdin, China) in absolute ethyl alcohol and diluted it with PBS. ML385, Nrf2 inhibitor, was purchased from TargetMol, United States. Astrocytes were transfected with FAM-labeled specific small interfering Connex43 RNA (si-Cx43) and negative control siRNA (si-NC) (GenePharma, China) by Lipofectamine^®^ 2000 reagent (Invitrogen, United States) under the manufacturer’s instructions. The sequences are listed as followed (sense/antisense, 5′-3′) (Yang et al., 2018), si-Cx43: ACAUCAUUGAGCUCUUCUATT/UAGAAGAGCUCAAUGA UGUTT; si-NC: UUCUCCGAACGUGUCACGUTT/ACGUGA CACGUUCGGAGAATT.

### Cell Viability Assay and Cytotoxicity Assessment by Lactate Dehydrogenase (LDH) Assay

Cell viability or LDH was evaluated with Cell Counting Kit-8 (CCK-8, Beyotime, China) or LDH cytotoxicity kit (Beyotime) following the manufacturer’s instructions. Astrocytes were plated into 96/24-well plate, with or without BM-MSCs coculture. The cells were treated with hemin for another 24 h. Then the experiment continued according to the instructions of the kit. The absorbance at 450 nm/490 nm was read with a microplate reader (BioTek, United States). The result was expressed as the percentage of the control group.

### ROS Assay and Measurement of MDA

Astrocytes were plated in 6-well plates, cocultured with or without BM-MSCs for 24 h. Reactive oxygen species assay kit (Beyotime) was applied to detected ROS accumulation following the manufacturer’s instructions. Five random fields were pictured using a fluorescence microscope (Leica, Germany). The contents of malondialdehyde (MDA) in the astrocytes were determined through thiobarbituric acid method using an MDA testing kit (Beyotime). Briefly, the cells plated in 6-well plates were homogenized in cell lysis buffer. The homogenate and working solutions were mixed (1:1) and centrifuged. The supernatant was suspended into TBA. After placing in a water bath for 15 min, the samples were centrifuged at 1000 *g* for 10 min. The optical density of the supernatants was measured at 532 nm with a microplate reader (BioTek).

### Total RNA Extraction and Quantitative Real-Time PCR (RT-PCR) Analysis

Total RNA was extracted from the peri-lesion area of the mice with Trizol reagent (Invitrogen, United States). Reverse transcriptase and Taq DNA polymerase (Yeasen Biotech Co., Ltd., China) were applied to reverse transcribed and amplified total RNA. The expression level of all transcripts was normalized to that of glyceraldehyde 3-phosphate dehydrogenase (GAPDH) mRNA. The mRNA relative expressions were normalized to control groups. All primer sequences were shown in Table 1.

**TABLE 1:** Primer sequences for qRT-PCR.

**Gene**	**Forward primer/Reverse primer (5′-3′)**
Cx43	GTGACAGAAACAATTCCTCCTG/ATTTTGCTCTGCGCTGTAATTC
Nrf2	TCCAGTCAGAAACCAGTGGAT/GAATGTCTGCGCCAAAAGCTG
HO-1	CAAGGAGGTACACATCCAAGCC/TACAAGGAAGCCATCACCAGCT
NQO1	TGGTGACATAATCCGACAAGAT/TTACCCACCTGAATGCCATAAT
SOD2	ACGCCACCGAGGAGAAGTACC/CGCTTGATAGCCTCCAGCAACTC
IL-6	TGGGACTGATGCTGGTGACA/ACAGGTCTGTTGGGAGTGGT
IL-10	CTGCTATGCTGCCTGCTCTTACTG/ATGTGGCTCTGGCCGACTGG
TNFα	TGATCGGTCCCAACAAGGA/TGCTTGGTGGTTTGCTACGA
GAPDH	GATGGTGAAGGTCGGTGTGA/TGAACTTGCCGTGGGTAGAG

*Cx43, connexin 43; Nrf2, NF-E2 related factor 2; HO-1, Heme oxygenase 1; NQO1, NAD(P)H: quinoneoxidoreductase1; SOD2, Superoxide Dismutase 2; IL-6, interleukin 6; IL-10, interleukin 10; TNFα, tumor necrosis factor α; GAPDH, glyceraldehyde 3-phosphate dehydrogenase.*

### Plasmid Constructs and Luciferase Reporter Gene Assay

We applied a luciferase reporter gene assay to confirm a direct link between Nrf2 and HO-1 in primary astrocytes. A fragment of HO-1, containing the promotor binding sequence (from -500 bp upstream to 100 bp downstream), was cloned to a luciferase reporter construct (GenePharma). Overexpressed Nrf2 plasmid (Nrf2) (GenePharma) was constructed by empty vector PCDNA 3.1 and transfected into astrocytes on 12-well plates, while PCDNA 3.1 was used as a negative control (NC). Furthermore, we constructed a mutation promotor of HO-1 at the same location with luciferase reporter, the mutation sites shown in Supplementary Figure S1a, and then Nrf2 plasmid and NC plasmid were transfected. 24 h later, the Dual-Glo Luciferase Reporter Assay kit (Promega) was applied to measure luciferase activity.

### TUNEL Staining

TUNEL staining (*In Situ* Cell Death Detection Kit, Roche, Germany) was used to detected cellular apoptosis. Astrocytes were seeded onto coverslips with or without BM-MSCs coculture and treated with 30 μM hemin for 24 h. After fixed in 4% paraformaldehyde for 20 min, permeated with 0.3% Triton X-100 for 15 min, coverslips were incubated with the reaction fluid in dark for 60 min at 37°C. The nuclei were stained with DAPI (1:5000, Beyotime) for another 5 min at RT in dark. The red nucleus staining of TUNEL positive cells was observed and analyzed by a confocal laser-scanning microscope (Leica). Five fields were randomly selected to calculate the ratio of TUNEL positive cells to total cells.

### Immunofluorescence

Brain cryosections and astrocytes coverslips were performed as previously described (Song et al., 2019), and immunostained with following primary antibodies: rabbit anti-Nrf2 polyclonal antibody (1:200, Santa Cruz Biotechnology, United States), rabbit anti-HO-1 polyclonal antibody (1:300, Abcam, United Kingdom), mouse anti-Cx43 monoclonal antibody (1:200, Invitrogen, United States), rabbit anti-GFAP polyclonal antibody (1:1000, Servicebio, China), mouse anti-VIM monoclonal antibody (1:500, Servicebio). The secondary antibodies used (1:500, Beyotime): Alexa Fluor 488 goat anti-rabbit IgG, Alexa Fluor 488 goat anti-mouse IgG, Alexa Fluor 555 donkey anti-mouse IgG, Alexa Fluor 555 donkey anti-rabbit IgG, Alexa Fluor 647 goat anti-mouse IgG, Nuclei were stained with DAPI (1:5000, Beyotime). The fluorescence images were observed and analyzed by a confocal laser-scanning microscope (Leica).

### Western Blotting Analysis

Brain tissues and astrocytes were treated according to experimental designs. WB was performed as previously described (Song et al., 2019). The following primary antibodies were used: rabbit anti-Nrf2 polyclonal antibody (1:500, Santa Cruz Biotechnology), rabbit anti-HO-1 polyclonal antibody (1:2000, Abcam), mouse anti-Connexin43 monoclonal antibody (1:1000, Invitrogen), rabbit anti-Cx43 polyclonal antibody (1:300, Sigma-Aldrich), rabbit anti-PKCα polyclonal antibody (1:1000, ABclonal Technology, China), rabbit anti-phospho-PKCα polyclonal antibody (1:1000, ABclonal Technology), rabbit anti-GFAP polyclonal antibody (1:1000, Servicebio), mouse anti-VIM monoclonal antibody (1:1000, Servicebio), rabbit anti-histone-H3 monoclonal antibody (1:1000, Cell Signaling Technology, United States), and mouse anti-GAPDH monoclonal antibody (1:2000, Servicebio). Enhanced chemiluminescence solution (Thermo Fisher Scientific) and Tanon Image (Shanghai, China) were used to detect the chemiluminescence signal. The relative intensity of the bands was measured by ImageJ 1.6.0 (NIH, United States).

### Co-immunoprecipitation of Cx43 and Nrf2

Immunoprecipitation was performed per the standard protocol as previously reported (Yang et al., 2018). Astrocytes were treated according to experiment designs, which were lysed with immunoprecipitation buffer and centrifuged at 12000 *g* for 10 min at 4°C. The supernatant was gathered for co-immunoprecipitation (Co-IP) and estimated for protein concentration. Then the lysates were pre-clearing with 20 μL washed protein A/G agarose (Santa Cruz Biotechnology) and centrifuged briefly to collect the supernatant. Lysates (1 mg protein) was incubated with 1 μg rabbit anti-Nrf2 monoclonal antibody (Santa Cruz Biotechnology), or 1 μg normal rabit IgG (Santa Cruz Biotechnology) with shaking for 12 h at 4°C. 20 μL protein A/G agarose was added to the complex and shaken for 4 h at 4°C. The agarose was then collected via centrifugation. Twenty μL 2^∗^loading buffer was added to the agarose bounding to the protein, boiled together at 99°C for 5 min, followed by WB analyzing with mouse anti-Cx43 monoclonal antibody (Invitrogen) to assess the connection between Cx43 and Nrf2 protein.

### Statistical Analysis

Data were expressed as mean ± standard deviation (SD) of at least three independent experiments. One-way ANOVA followed by Kruskal–Wallis test was applied for comparisons among several groups. Comparisons between two groups were made by Student’s *t* test. A *p*-value of < 0.05 was taken to indicate statistical significance. Statistical calculations were performed using SPSS 20.0 (SPSS, Chicago, IL, United States). GraphPad Prism 5 (CA, United States) was used to draw statistical charts.

## Results

### BM-MSCs Isolation, Identification, and Transplantation

BM-MSCs were isolated from the femoral and tibial bone marrow of adult male SD rats and maintained in culture for several passages. The cultured cells demonstrated a typical spindle-shaped morphology (Figures 1A,B). Before intracranial transplantation, cells were characterized, and flow cytometry analysis confirmed that the cells at transplantation were positive for CD90 (97.90%) and CD29 (99.98%), and had low expression of CD34 (0.59%) and CD45 (1.01%; Figure 1C), which was highly consistent with previous studies (Dominici et al., 2006). For cell tracking, red fluorescent (PKH-26)-positive cells, indicating transplanted cells, were located in the hemisphere ipsilateral to the ICH 3 days after transplantation (Figures 1E,F).

### BM-MSCs Transplantation Attenuated Brain Water Content, Reduced Hematoma Volume, and Improved Neurological Behavior Impairment After ICH

**FIGURE 2:**
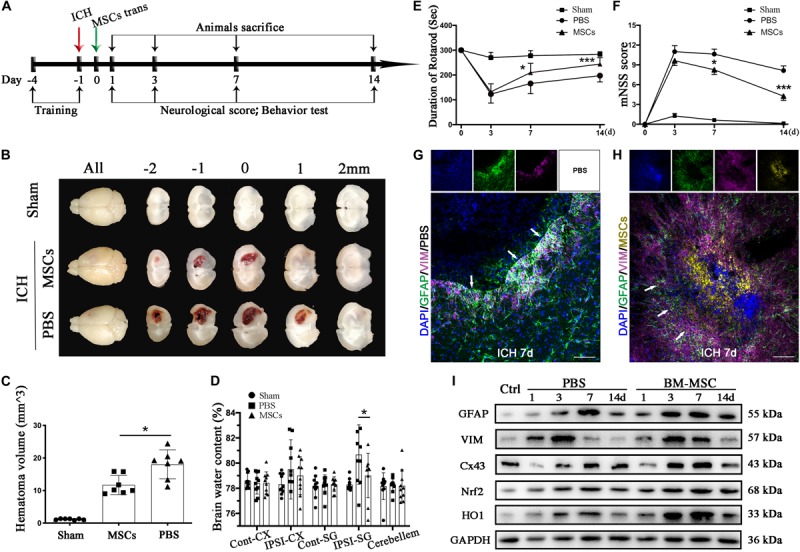
BM-MSCs transplantation attenuated brain water content, reduced hematoma volume, improved neurological outcomes, and promoted astroglial mesenchymal phenotype switching of astrocytes after ICH. **(A)** Diagram *in vivo* experiments. **(B)** Coronal sections of brain tissues after 3 days post-transplantation. **(C)** The volume of ICH in BM-MSCs and PBS treated Mice after 3 days post-transplantation (*n* = 7). **(D)** Brain water content at 3 days post-transplantation (*n* = 10). **(E,F)** BM-MSCs improved neurological outcomes both in the rotarod test and mNSS (*n* = 10). All data are displayed as means ± SD. The difference between groups was analyzed using One-way ANOVA test. *p < 0.05, **p < 0.01, ***p < 0.001. **(G)** Immunofluorescence staining for VIM (purple) and GFAP (green) in ICH mouse brain at 7 days post-PBS transplantation. Bar = 100 μm. GFAP was strongly expressed in reactive astrocytes, and VIM was observed around the lesion area 7 days after ICH. Glial scar (white arrows) could be seen around the hematoma. **(H)** Immunofluorescence staining for VIM (purple) and GFAP (green) in ICH mouse brain at 7 days with BM-MSCs (yellow) transplantation. Bar = 100 μm. After the transplantation of BM-MSCs, VIM maintained high expression, whereas GFAP was kept at a relative low level. **(I)** Western blotting analysis of Cx43, GFAP, VIM, Nrf2 and HO-1 expression in ICH mouse brain of control, PBS, and BM-MSCs treatment at 1, 3, 7, and 14 days. The results of densitometric analysis of the bands were shown in Supplementary S1.

Brain water content was measured to explore the effects of BM-MSCs treatment on ICH-induced brain edema. No significant differences were noted in the contralateral cortex, contralateral basal ganglia, cerebellum, or ipsilateral cortex in the three groups (Figure 2D). In the ipsilateral basal ganglia, the water content in the PBS group was significantly higher than that in the sham group, and the water content in the MSCs group was significantly lower than that in the PBS group (*p* < 0.05). BM-MSCs transplantation also ameliorated ICH volume on day 3, compared with the PBS group (*p* < 0.05; Figures 2B,C). We further tested the neurological outcomes at 3, 7, and 14 days after BM-MSCs transplantation, using the rotarod test and modified neurological severity score (mNSS). The results showed that neurological deficits were significantly reduced after BM-MSCs transplantation at 7 and 14 days (*p* < 0.05 and *p* < 0.001, respectively; Figures 2E,F), when compared to the PBS group.

### BM-MSCs Transplantation Promoted Astrocytes Transformation of Mesenchymal Phenotypes, and Enhanced Nrf2 and HO-1 Expression After ICH *in vivo*

Astrocytes express the main intermediate filament (IF) protein subtypes of glial fibrillary acidic protein (GFAP), together with vimentin (VIM), nestin, and diamine (Hol and Pekny, 2015). VIM and GFAP are the two IF components associated with astrocyte activation and reactive gliosis in response to CNS injury, both in the early and late stages; thus, increased expression of GFAP and VIM is widely used as a marker of reactive astrocytes (Pekny and Pekna, 2014; Hol and Pekny, 2015; Kamphuis et al., 2015). We performed immunofluorescence staining and WB analysis to detect the expression pattern of GFAP and VIM in ICH. As shown in Figure 2G, GFAP was strongly expressed in reactive astrocytes, and low levels of VIM were observed around the lesion area 7 days after ICH. Glial scars with high expression of GFAP were visualized around the hematoma. After the transplantation of BM-MSCs, VIM maintained high expression at 7 days, whereas GFAP remained at a relatively low level, compared with the ICH-PBS group (Figure 2H). We also found that Nrf2 and HO-1 expression increased after ICH, but after BM-MSCs transplantation, Nrf2 and HO-1 expression increased significantly compared to the PBS-ICH group (*p* < 0.05; Figure 2I and Supplementary Figures S1a-e).

### BM-MSCs Co-culture Enhanced the Resistance of Astrocytes to Hemin Neurotoxicity, Downregulated ROS and MDA Accumulation, and Regulated mRNA Expression of Cytokines *in vitro*

**FIGURE 3:**
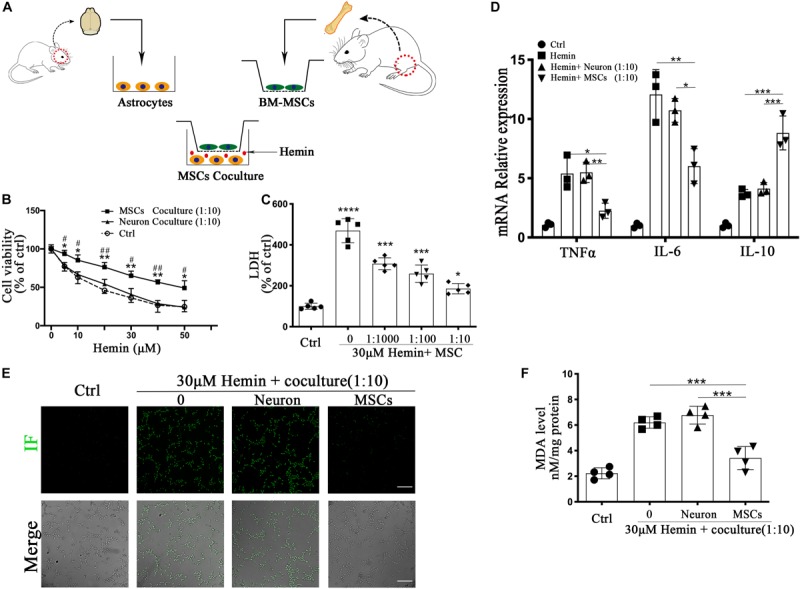
BM-MSCs coculture protected astrocytes from neurotoxicity induced by hemin. **(A)** Schema of primary cells and co-culture of rat BM-MSCs and mouse astrocytes. **(B)** Astrocytes were exposed to 0, 5, 10, 20, 30, 40, 50 μM hemin for 24 h with or without BM-MSCs/Neuron coculture, then the cell viability was evaluated by CCK-8 (*n* = 5). **(C)** Astrocytes were exposed to 30 μM hemin with or without BM-MSCs for 24 h, and the cell death was evaluated by LDH releasing assay (*n* = 5). **(D)** mRNA expression of TNFα, IL-6, and IL-10 was checked, and the relative expression of the mRNA was normalized to control (*n* = 3). **(E)** After DCFH-DA probes were loaded, the intracellular ROS was observed using fluorescent microscopy, bar = 400 μm. **(F)** The contents of MDA in the astrocytes were determined (*n* = 4). All data are displayed as means ± SD. The difference between groups was analyzed using One-way ANOVA test. *p < 0.05, **p < 0.01, ***p < 0.001, ****p < 0.0001, compared with control (0 μM); ^#^*p* < 0.05, ^##^*p* < 0.01, compared with Neuron coculture (1:10).

Through *in vivo* studies, we concluded that astrocytes responded to BM-MSCs; we therefore established an ICH model *in vitro* by exposing primary astrocytes to hemin, with or without BM-MSCs or neurons coculture to hemin. The schematic diagram is shown in Figure 3A. We evaluated astrocyte viability and death by the CCK-8 and LDH-releasing assays, after hemin exposure, with or without BM-MSCs or neurons coculture (at a ratio of 1:10). The results showed that the cell viability increases with the increase in hemin dosage (Figures 3B,C; *p* < 0.05). The neurotoxicity of hemin is predominatly due to the production of ROS. Therefore, we explored whether BM-MSCs coculture could protect astrocytes against hemin neurotoxicity, by blocking the accumulation of intracellular ROS accumulation. Our experimental results confirmed this hypothesis that BM-MSCs coculture significantly reduces ROS accumulation (Figure 3E). Since the half-life of ROS is short, the examination of oxidative productions like malondialdehyde (MDA) was checked further. The levels of lipid peroxidation (MDA) were significantly decreased in the BM-MSC coculture group, compared to the no coculture and neuron coculture group (*p* < 0.001 and *p* < 0.001, respectively; Figure 3F). We further analyzed cytokine mRNA expression using qRT-PCR and found that TNFα and IL-6 decreased significantly after BM-MSCs coculture (*p* < 0.05, *p* < 0.01 respectively), but IL-10 increased significantly, when compared to the no coculture group (*p* < 0.001; Figure 3D).

### BM-MSCs Coculture Activated Astrocytes Underwent a Transformation in Mesenchymal Phenotypes

**FIGURE 4:**
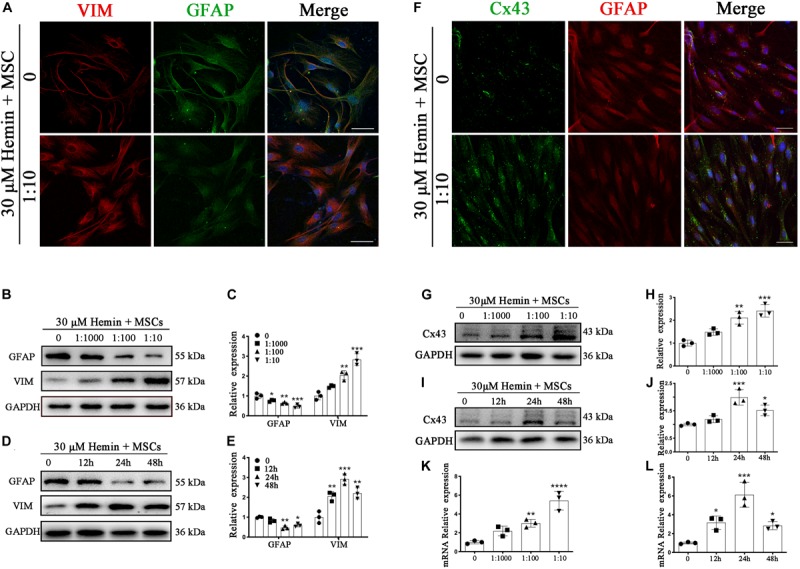
BM-MSCs coculture induced an epithelial-mesenchymal switch, and induced Cx43 upregulation in astrocytes. **(A)** Immunofluorescence staining of astrocytes for VIM (red) and GFAP (green). The cell nuclei were counterstained with DAPI(blue). Bar = 25 μm. **(B–E)** Western blotting analysis of GFAP and VIM in astrocytes exposure to 30 μM hemin, cocultured with BM-MSCs for indicated times and doses. **(F)** Immunofluorescence staining for Cx43 (green) and GFAP (red) in astrocytes treated with or without BM-MSCs coculture. The cell nuclei were counterstained with DAPI (blue). Cx43 was expressed on the cell membrane where astrocytes contact, after coculture with BM-MSCs, Cx43 expression was increased. Bar = 25 μm. **(G–J)** Western blotting analysis of Cx43 and GFAP in astrocytes exposure to 30 μM hemin, cocultured with BM-MSCs for indicated times and doses. **(K,L)** Cx43 mRNA expression of Cx43 in astrocytes exposure to 30 μM hemin, cocultured with BM-MSCs for indicated times and doses. Expressions were normalized against the internal reference GAPDH. The fold change values were calculated by normalizing to control samples. All data are displayed as means ± SD (*n* = 3). The difference between groups was analyzed using One-way ANOVA test. *p < 0.05, **p < 0.01, ***p < 0.001, compared with control (0 h or 0 μM).

With respect to the expression of IF proteins, when astrocytes are placed in culture, the differentiation process of cultured astrocytes from a newborn mouse brain closely follows that of astrocytes in the developing brain (Galou et al., 1996). To confirm that astroglial mesenchymal phenotype switching occurs *in vitro*, immunofluorescence staining was performed to detect GFAP and VIM in astrocytes with or without BM-MSCs coculture. The results (Figure 4A) confirmed a trend to astroglial mesenchymal phenotype switching. WB analysis was used to confirm the protein expression of GFAP and VIM. Astrocytes were cocultured with BM-MSCs at concentrations of 0, 1:1000, 1:100, and 1:10 for 24 h or at a concentration of 1:10 for 0, 12, 24, and 48 h. The WB results showed that GFAP expression decreased significantly with increases in both coculture concentration and coculture time (*p* < 0.05; Figures 4B–E). The expression of VIM vary from that of GFAP. These results supported astroglial mesenchymal phenotype switching following the activation of astrocytes induced by BM-MSCs coculture.

### BM-MSCs Induced Cx43 Upregulation *in vivo* and *in vitro*

Intercellular GJs, based on Cx43, are essential for retaining astrocyte homeostasis. Once ICH occurs, homeostasis is disrupted. To evaluate whether Cx43 expression is affected by ICH, immunofluorescence staining, WB, and qRT-PCR were performed. As demonstrated (Figure 2I), protein expression of Cx43 was downregulated in the mouse brain at 1 day (*p* < 0.01), elevated from 3 days, peaked at 7 days, and returned to normal levels at 14 days. After BM-MSC transplantation, protein expression upregulated significantly at 3 and 7 days (*p* < 0.01 and *p* < 0.001, respectively). Through immunofluorescence staining (Figure 4F), we found Cx43 expression not only on the cell membrane where astrocytes were contacted, but also in astrocyte cytoplasm. Cx43 protein and mRNA expression increased after coculture with BM-MSCs. The WB results showed that Cx43 protein expression was significantly increased in association with an increase in MSC coculture concentration (*p* < 0.05; Figures 4G,H) and reached the highest expression at 24 h (*p* < 0.001; Figures 4I,J). The qRT-PCR analysis also revealed that Cx43 mRNA expression increased synchronously with protein levels (Figures 4K,L). These results indicate that BM-MSC coculture increased Cx43 mRNA transcription and induced Cx43 protein upregulation.

### BM-MSCs Coculture Induced Nrf2 Nuclear Translocation and Enhanced HO-1 Expression

**FIGURE 5:**
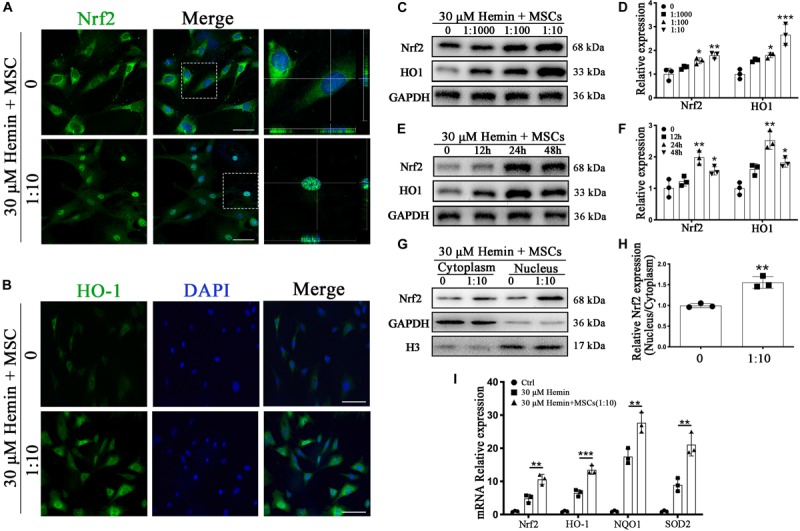
BM-MSCs coculture induced Nrf2 upregulation and promoted its nuclear translocation in astrocytes. **(A)** Immunofluorescence staining of astrocytes with Nrf2 (green). The cell nuclei were counterstained with DAPI (blue). BM-MSCs coculture treatment triggered Nrf2 nuclear translocation in astrocytes exposure to 30 μM hemin. Bar = 25 μm. **(B)** Immunofluorescence staining of astrocytes with HO-1 (green). The cell nuclei were counterstained with DAPI (blue). BM-MSCs coculture treatment increased HO-1 expression in astrocytes exposure to 30 μM hemin. Bar = 25 μm. **(C–F)** Western blotting analysis of Nrf2 and HO-1 expression in astrocytes exposure to 30 μM hemin, cocultured with BM-MSCs for indicated times and doses. **(G)** Western blotting analysis of cytoplasmic and nucleus extraction samples from astrocytes, cocultured with BM-MSCs or not, with Nrf2 antibody. GAPDH and H3 were used as a loading control for cytoplasmic and nucleus protein, respectively. **(H)** The histogram showing the results of densitometric analysis of nucleus/cytoplasmic Nrf2 expression in astrocytes cocultured with BM-MSCs or not. **(I)** mRNA expression of Nrf2, HO-1, NQO1, SOD2 in astrocytes with or without BM-MSCs coculture exposed to 30 μM hemin for 24 h. The relative expression of the mRNA was normalized to control. The results of densitometric analysis of the bands were plotted into histogram. All data are displayed as means ± SD (*n* = 3). The difference between groups was analyzed using One-way ANOVA test. Comparisons between two groups were made by Student’s *t*-test. *p < 0.05, **p < 0.01, ***p < 0.001, compared with control (0 h or 0 μM).

Nrf2 is a critical transcription factor, regulating antioxidant reaction against ROS, and is disrupted after ICH. To explore the changes after ICH, we tracked the expression of Nrf2 and HO-1 by immunofluorescence staining as well as WB analysis. Luciferase gene reporter indicated the fluorescence activity of astrocytes with overexpressed Nrf2 plasmid were significantly increased (*p* < 0.001), but this decreased significantly in the interference group (*p* < 0.05), compared to the NC plasmid (Supplementary Figures S2a,b); this suggested that Nrf2 and HO-1 were indeed associated in astrocytes. Immunofluorescence staining revealed that astrocytes cocultured with BM-MSCs after hemin exposure were associated with increased HO-1 staining and Nrf2 nuclear translocation, compared to those not cocultured (Figures 5A,B). The protein expression of HO-1 and Nrf2 also increased in association with an increase in BM-MSC coculture concentration, reaching the highest expression at 24 h and reducing at 48 h (*p* < 0.05; Figures 5C–F). We further examined Nrf2 expression in nuclear and cytoplasmic samples, corroborating that the Nrf2 nucleus to cytoplasm ratio in astrocytes with BM-MSC coculture was significantly higher than that seen in the control group (*p* < 0.01; Figures 5G,H). Then, we further evaluated the effect of BM-MSCs on mRNA expression of Nrf2 and its antioxidant enzymes, independent of HO-1. Results were clear with regards to Nrf2 and HO-1 protein expression (Figure 5I).

### Nrf2 Inhibition Counteracted BM-MSCs Coculture Induced Antioxidation

**FIGURE 6:**
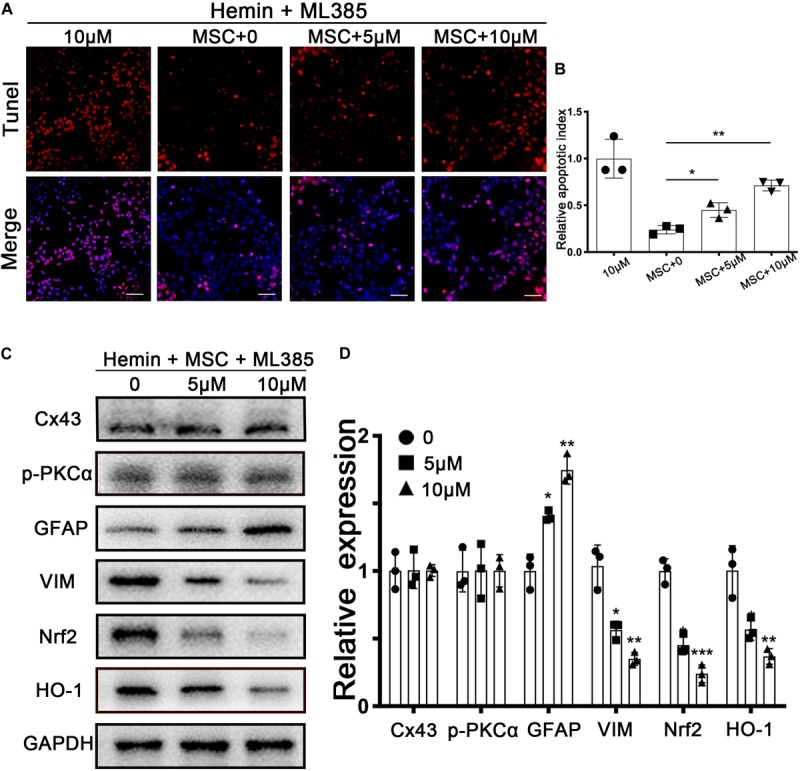
Nrf2 inhibitor ML385 counteracted BM-MSCs coculture induced antioxidation and GFAP/VIM switch. Astrocytes were administrated with or without ML385 for 6 h, then followed by 30 μM hemin incubation, with or without BM-MSCs coculture treatment. **(A)** TUNEL staining (red) was used to mark apoptotic cells. Bar = 50 μm. **(B)** The results of the apoptosis rate and densitometric analysis of the bands were plotted into a histogram of five randomly fields. **(C,D)** Western blotting analysis of Cx43, GFAP, VIM, Nrf2, HO-1 protein expression was examined. The relative expression was normalized to control. All data are displayed as means ± SD (*n* = 3). The difference between groups was analyzed using One-way ANOVA test. *p < 0.05, **p < 0.01, ***p < 0.001, compared with control (ML385 0 μM).

Nrf2 has transcriptional activity when translocated into nuclei, after which it regulates its downstream genes ARE, in response to antioxidants. ML385, widely accepted as an Nrf2 inhibitor, was used to downregulate Nrf2 expression; as per previous studies, we chose 5 and 10 μM as the ML385 concentration in our *in vitro* experiment (Jiao et al., 2019; Zhang et al., 2019). Using the above *in vitro* model, we found that the ratio of TUNEL-positive cells was significantly increased, in a dose-dependently manner, after ML385 administration (*p* < 0.05 and *p* < 0.01, respectively; Figures 6A,B). The upregulation of VIM and downregulation of GFAP were reversed after ML385 administration (*p* < 0.05; Figures 6C,D). These results supported the role of Nrf2 signaling in the antioxidant and astroglial mesenchymal phenotype switching of astrocytes associated with BM-MSCs coculture.

### Knockdown of Cx43 Cut Down Nrf2 Nucleus Translocation and Restrained Phosphorylation of PKCα

**FIGURE 7:**
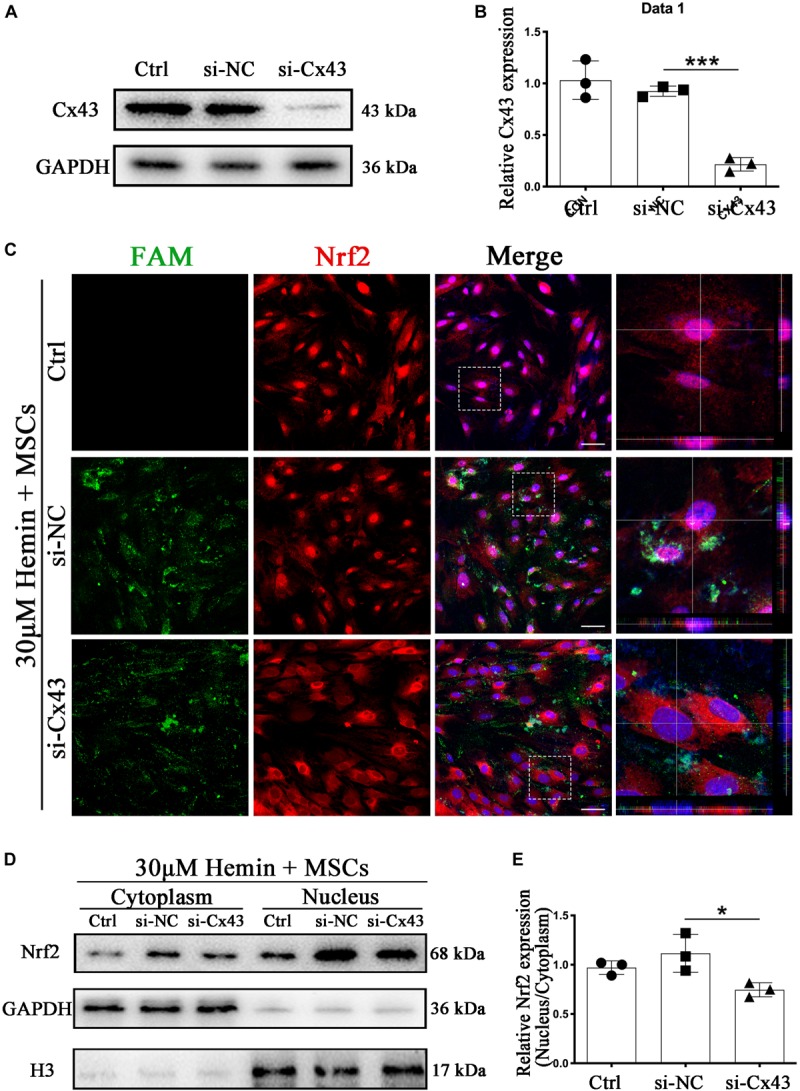
Cx43 knockdown suppressed BM-MSCs-induced Nrf2 nuclear translocation. **(A,B)** Western blotting analysis of Nrf2 expression in control, si-NC, si-Nrf2 transfected astrocytes. **(C)** Immunofluorescence staining of control, si-NC, si-Cx43 treated astrocytes with anti-Nrf2 (red). The siRNA was labeled with fluorophore FAM (green) to show the transfected cells. The cell nuclei were counterstained with DAPI (blue). Bar = 50 μm. **(D)** Western blotting analysis of cytoplasmic and nucleus extraction samples from control, si-NC, si-Cx43 transfected astrocytes with anti-Nrf2. GAPDH and H3 were used as a loading control for cytoplasmic and nucleus protein, respectively. **(E)** The histogram showing the results of densitometric analysis of nucleus/cytoplasmic Nrf2 expression in control, si-NC, si-Cx43 transfected astrocytes. All data are displayed as means ± SD (*n* = 3). The difference between groups was analyzed using One-way ANOVA test. *p < 0.05, ***p < 0.001.

Subsequently, we turned our attention to explore whether Cx43 upregulation was closely related to Nrf2 nuclear translocation. To confirm whether Nrf2 nuclear translocation was triggered by Cx43 upregulation, we performed the si-RNA interference experiment. finding more than 80% of Cx43 was restrained by si-Cx43 (*p* < 0.001; Figures 7A,B). With immunofluorescence staining, Nrf2 was mainly located in the cytoplasm in si-Cx43 transfected astrocytes with BM-MSCs coculture. In contrary, Nrf2 nuclear translocation was evident in control and si-NC transfected astrocytes with BM-MSCs coculture, as shown with immunostaining (Figure 7C) and WB analysis (*p* < 0.05; Figures 7D,E). These results suggest that Cx43 upregulation might be one of the upstream events of Nrf2 nuclear translocation. Some evidence suggests that PKCα, an upstream of Nrf2, can regulate Nrf2 activation (Huang et al., 2000; Niture et al., 2010); therefore, we explored whether Cx43 knockdown would lead to the changes in PKCα expression. WB analysis showed that phosphorylation of PKCα increased after BM-MSCs coculture, and it was significantly inhibited after si-Cx43 administration, compared to the si-NC group (*p* < 0.05; Supplementary Figure S3).

### Cx43 and Nrf2 Are Connected

**FIGURE 8:**
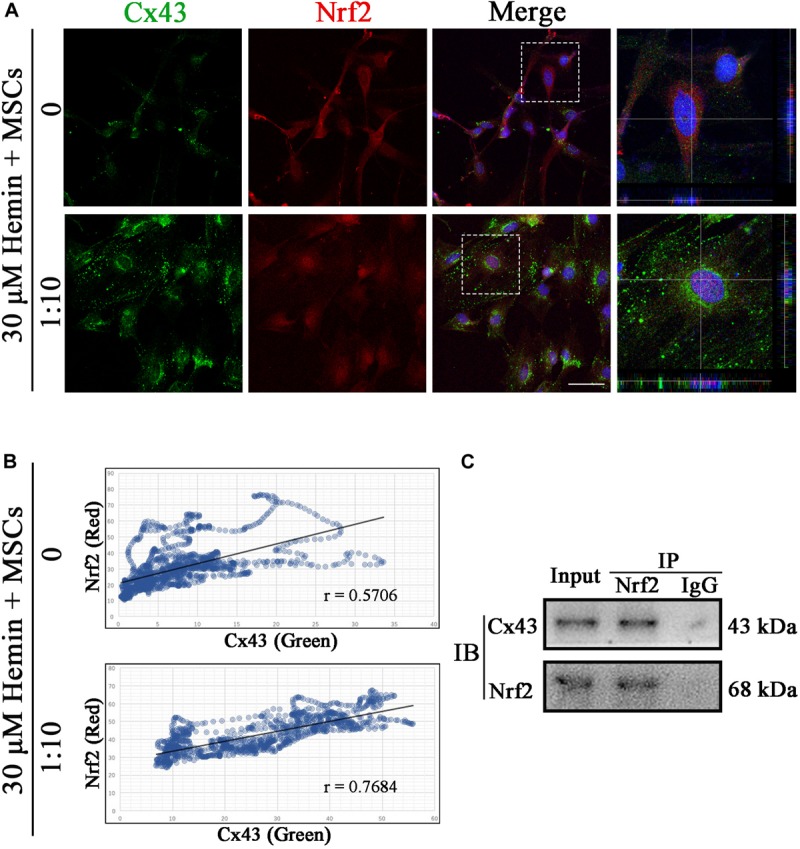
Cx43 and Nrf2 were co-localized and physically associated. **(A)** Immunofluorescence double staining for Cx43 (green) and Nrf2 (red) in astrocytes exposed to 30 μM hemin, with or without BM-MSCs coculture treatment. The cell nuclei were counterstained with DAPI (blue). Both proteins were co-localized at the cell–cell contact in untreated astrocytes, and Nrf2 was located in the cytoplasm at this time. After co-culture with BM-MSCs, the expression of Cx43 increased and Nrf2 accumulated in the nuclei. Bar = 25 μm. **(B)** Pearson correlation analysis was used to check the relationship between Cx43 and Nrf2. In the scatter plot, the symbol *r* represented the Pearson’s correlation coefficient (*n* = 3). **(C)** Western blotting analysis of Nrf2 immunoprecipitated samples using mouse anti-Cx43 monoclonal antibody. Cx43 was detected in the immunoprecipitated complex.

To detect whether there was a connection between Cx43 and Nrf2, we performed immunofluorescence staining and co-immunoprecipitation (Co-IP). As shown in Figure 8A,B, Cx43 and Nrf2 co-localize at Gjs and cytoplasm (*r* = 0.5706). Strikingly, after coculture with BM-MSCs, the fluorescence of Cx43-Nrf2 complex was stronger (*r* = 0.7684), resulting in Nrf2 nuclear translocation. With Nrf2 immunoprecipitation, Cx43 was detected in the precipitated complex (Figure 8C). In summary, both immunofluorescence staining and Co-IP suggested that there was a direct or indirect connection between Cx43 and Nrf2, which was enhanced after BM-MSCs coculture.

## Discussion

Astrocytes are the major glial cells in the CNS and outnumber neurons by several folds (Hol and Pekny, 2015). As an important component of the neuro-glial system, astrocytes have key roles in maintaining CNS homeostasis, including neurotrophic and structural supporting functions, maintenance of the extracellular environment, stabilization of cell–cell communications, and anti-oxidative stress functions (Ransom and Ransom, 2012). Astrocyte activation is a usual response to injury to the CNS, the function role of which remains controversial (Liu et al., 2014). In glial scars, reactive astrocytes would express a wide range of molecules that inhibit axonal regeneration (McKeon et al., 1991). However, glial scars also have a positive role in isolating injured sites from healthy tissues, forming a physical and chemical barrier, and preventing inflammatory waves leading to uncontrolled tissue damage (Faulkner et al., 2004). In addition, reactive astrocytes might produce neurotrophic factors, and protect neurons from ischemic injury (Hansson and Ronnback, 2003), the key challenge in the treatment of ICH is to therefore understand how to magnify the advantages and minify the disadvantage of reactive astrocytes. GFAP and VIM are the two IF components associated with astrocyte activation and reactive gliosis, and particular transitions are observed following CNS injury, which have been widely utilized as a marker of reactive astrocytes (Galou et al., 1996; Pekny and Pekna, 2014; Hol and Pekny, 2015; Kamphuis et al., 2015). The astrocytes of VIM-GFAP double knockout mice are obviously deficient in cytoplasmic IFs, and characterized by decreased reactive glial degeneration and a lack of characteristic hypertrophy of astrocyte processes, after CNS injury (Wilhelmsson et al., 2004).

MSCs are believed to pass through the blood brain barrier, migrate to the damage boundary sites, and reduce apoptosis of astrocytes (Zhao et al., 2002; Tang et al., 2014). Early and sustained beneficial effects of MSCs secreting bioactive factors with neurotrophic/immunoregulatory potential on functional and structural damage have been observed (Borlongan et al., 2004). Animals presented a natural ability to recover from behavioral deficits gradually after ICH injury without cell therapy, but 7 days after the transplantation of BM-MSCs, the cognitive and motor function of the brain-injured mice were facilitated significantly. The difference was more significant on 14 days after the transplantation of BM-MSCs (Bedini et al., 2018). Although there was no significant difference was found in the water content of the Ipsi-Cx, brain edema still showed a downward trend in the BM-MSC transplantation group. This might be because our collagenase injection mimics the human hypertensive ICH model, centered on the basal ganglia. These results suggested that BM-MSCs transplantation could ameliorate brain injury (including neurological brain edema, behavioral impairment, and hematoma volume) after ICH.

MSCs therapy has been reported to modulate microglial activation toward a pro-regenerative function after stroke, by maintaining the resting microglial phenotype, or by controlling the microglial activation (Wei et al., 2012; Yan et al., 2013). Donega et al. (2014) also reported that human-MSCs treatment reduced GFAP expression and glial scars formation. During our *in vivo* study, we observed an increase in VIM expression after ICH. As mentioned earlier (Yang et al., 2018), there were temporal and spatial differences in the expression of GFAP and VIM in our ICH model. After BM-MSCs transplantation, VIM increased early and remained elevated for a long time, and the GFAP was suppressed at a relatively low level. In our previous studies (Yang et al., 2018), we have proposed that, following ICH, astrocytes might undergo dedifferentiation. Dedifferentiation might be a mechanism of astrocytes to dealing with injury, promoting astrocytes anti-apoptosis and activation. Lynch et al. (2013) have revealed that VIM is essential for proper cell spreading, which could regulate adhesion and focal contact size, and control cell adhesion strength (Hol and Pekny, 2015). This might help to isolate injured sites from healthy tissues. After BM-MSCs transplantation, de-differentiation is enhanced, VIM expression is increased, apoptosis is inhibited, hypertrophy is regulated, and ultimately formation of the glial scar is inhibited. Our *in vitro* experiments showed that GFAP was downregulated, but VIM was upregulated, in cultured astrocytes with BM-MSCs coculture after exposure to hemin. We found a mismatch between the *in vivo* and *in vitro* dynamics of astrocyte IF expression, which may be due to differences between the *in vivo* and *in vitro* models. In the *in vivo* model, we used collagenase to induce ICH, while in the *in vitro* model, we used hemin, which is the purified form of natural heme *in vitro*. We also found that astrocytes associated with BM-MSCs coculture were resistant to neurotoxicity *in vitro*, by downregulating the pro-inflammatory cytokines TNF-α and the inflammatory cytokines IL-6, and upregulating the anti-inflammatory cytokines IL-10. To demonstrate the specific ability of BM-MSCs to regulate cytokines, we added a negative control group (neuron coculture, at a ratio of 1:10) to our *in vitro* experiments, to prove their antioxidant capacity. Finally, The results paralleled with Aggarwal’s research (Aggarwal and Pittenger, 2005). IL-10 is well-known to suppress the pro-inflammatory phenotype of astrocytes and microglia (Ziebell and Morganti-Kossmann, 2010). Although the observation that the neuroprotective effect of BM-MSCs may occur *in vivo* is consistent with primary astrocytes cocultured with BM-MSCs *in vitro*, data *in vitro* were obtained through a transwell system, which could strongly suggest that BM-MSCs protect astrocytes from apoptosis through regulating anti-inflammatory, pro-inflammatory and inflammatory cytokines rather than through cell–cell contact (Supplementary Figure S4).

Gj communication is considered essential in many biological processes, such as control of cell proliferation, differentiation and proper migration, embryonic development, and wound healing et al. (Reaume et al., 1995). Intercellular Gjs based on Cx43 are the main cell adhesions in astrocytes. Zhang et al. (2015) have also shown that pro-inflammatory cytokines can promote Cx43 degradation via ubiquitin-proteasome. Rucker-Martin et al. (2006) also showed the distribution of Cx43 was affected by fibrosis, destroying the cytoskeleton network and adhesion proteins, which were related to normal Cx43 organization and function. In our research, astrocytes apoptosis following ICH led to disruption of homeostasis simultaneously with Cx43 downregulation, finally leading to Cx43 conformational changes, channel closure, and degradation. After BM-MSCs transplantation, the Cx43 protein expression was significantly higher at 3 and 7 days when compared to the PBS group. Similar results were obtained in our *in vitro* experiments, where coculture of astrocytes with BM-MSCs was associated with stronger Cx43 expression and lower GFAP expression, which correlated with the concentration of BM-MSCs. Based on this work, we propose that the down-regulation of Cx43 in activated astrocytes after ICH might result from the production of inflammatory cytokines, whereas after BM-MSCs transplantation, the downregulation of Cx43 was restrained.

Nrf2 is a key transcription factor regulating antioxidant defense. Once stimulated by oxidative stress, Nrf2 escapes from Keap1, transfers to the nucleus, and results in the upregulation of ARE-mediated gene expression (Huang et al., 2000; Wang et al., 2012; Liu et al., 2015). The Nrf2-ARE signaling pathway is considered a multiple-organ protective agent, playing a vital role in several CNS diseases, including subarachnoid hemorrhage (SAH), cerebral ischemia, traumatic brain injury and cerebral hemorrhage (Shih et al., 2005; Chen et al., 2011; Yin et al., 2015). The Nrf2 pathway was activated in astrocytes to protect themselves and their adjacent neurons from oxidative damage (Wang et al., 2019). We also found coculture with BM-MSCs increased expression of Nrf2 and HO-1 RNA and protein in astrocytes, compared to the control and untreated groups. Application of Nrf2 inhibitors to block upregulation and translocation resulted in no significant change in the expression of Cx43 and p-PKCα, while both anti-apoptotic ability and astroglial-mesenchymal phenotype switching were restrained. Wang et al. (2007) found that Nrf2 knockout impairs neurological function after ICH. Our findings suggest that Nrf2 is a positive regulator of anti-apoptosis and participates in reactive astrogliosis, and the anti-inflammatory factors released by BM-MSCs trigger increased Nrf2 translocation.

Several studies have described the importance of Cx43 in antioxidant stress. Inhibition of Cx43 degradation could protect cardiomyocytes exposed to hydrogen peroxide, by inhibiting oxidative stress (Lee et al., 2018). Chen et al. (2017) revealed that Cx43 could regulate Nrf2/ARE signaling to resist oxidative stress injury in glomerular mesangial cells. Le et al. (2014) showed that Gjs mediated by Cx43 in astrocytes have a positive effect on resistance to oxidative stress. Based on the above, we explored whether the regulation of Cx43 would result in the changes to Nrf2 expression after ICH. We found that knockdown of Cx43 prevented Nrf2 nuclear translocation and PKCα phosphorylation. PKC is one of several protein kinases able to modulate Nrf2 activity; furthermore, phosphorylation of Nrf2 serine 40 can lead to the escape or release of Nrf2 from Keap1, allowing it to translocate to the nucleus, and bind to the ARE that leads to coordinated activation of gene expression (Huang et al., 2000, 2002). Therefore, in combination with our experimental results, we believe that Cx43 might cause Nrf2 to separate from Keap1 and transfer into the nucleus, through phosphorylation of PKCα, to play a role in antioxidant stress.

In addition, we applied double immunostaining and Co-IP to confirm the connection between Cx43 and Nrf2. These results supported our hypothesis that elevated Cx43 promoted Nrf2 nuclear translocation and its phase II enzyme expression. After BM-MSCs transplantation, this phenomenon was enhanced. The potential mechanisms are shown in Supplementary Figure S4. Whether Cx43 would be directly bound to these regulatory genes, or mediated by other proteins besides PKCα, still needs further confirmation. A limitation of our study was that the implanted BM-MSCs were not homologous with the host, and the host tissue immunological response could also damage to the BM-MSCs. Further studies are required to explore how to best ensure the activity of implanted cells.

## Conclusion

ICH is a severe insult to the CNS, and astrocytes are the primary defense system. Astrocyte activation is a double-edged sword, but BM-MSC transplantation could help improve the anti-apoptotic ability of reactive astrocytes, trigger GFAP/VIM switch, and inhibit the final formation of the glial scar. Following the secretion of bioactive factors from BM-MSCs, Cx43 retards degradation, which helps liberate Nrf2 from Keap1 and allows its nuclear translocation, promoting phase II detoxification enzyme genes involved in antioxidant stress and anti-apoptosis. Astrocyte Cx43/Nrf2 interactions after BM-MSC transplantation may provide an important therapeutic target in the management of ICH.

## Data Availability Statement

The datasets generated for this study are available on request to the corresponding author.

## Ethics Statement

The animal study was reviewed and approved by The Animal Care and Use Committee of Ruijin Hospital, Shanghai Jiao Tong University.

## Author Contributions

XC, HS, and LB designed the research and wrote the manuscript. XC and HL analyzed the results. XC, HL, and ZX prepared and completed the BM-MSCs and astrocytes’ isolation and culture. XC, HS, YS, CX, and ZZ helped complete the animal experiment and collected the data and performed the statistical analysis. YS, BW, and YY provided useful suggestions on experiment design and reviewed the manuscript. YS and LB provided funds collection. G-YY, QS, and LB assisted with reviewing, editing the manuscript, and provided expertise and feedback.

## Conflict of Interest

The authors declare that the research was conducted in the absence of any commercial or financial relationships that could be construed as a potential conflict of interest.

## Supplementary Material

FIGURE S1 Western blotting analysis of Cx43, GFAP, VIM, Nrf2 and HO-1 expression in ICH mouse brain of control, PBS, and BM-MSCs treatment at 1, 3, 7, and 14 days. **(A–E)** The results of densitometric analysis of the bands. All data are displayed as means ± SD (*n* = 3). The difference between groups was analyzed using One-way ANOVA test. **p* < 0.05, ***p* < 0.01, ****p* < 0.001, compared with control.

FIGURE S2 The diagram and results of luciferase activity analysis between Nrf2 and HO-1 in primary astrocytes. **(a)** The HO-1 wildtype group (WT) and the HO-1 mutation group (Mut) were set up to verify our experiments. Three mutation sites were set at – 303 ∼ – 298 bp, – 138 ∼ – 133 bp, and – 2 ∼3 bp, respectively (from −500 bp upstream to 100 bp downstream). **(b)** All data are displayed as means ± SD (*n* = 4). The difference between groups was analyzed using One-way ANOVA test. **p* < 0.05, ****p* < 0.001, compared with control.

FIGURE S3 Cx43 knockdown suppressed BM-MSCs-induced p-PKCα expression. **(a,b)** Western blotting analysis of p-PKCα and PKCα expression in control, si-NC, si-Nrf2 transfected astrocytes. All data are displayed as means ± SD (*n* = 3). The difference between groups was analyzed using One-way ANOVA test. **p* < 0.05, ***p* < 0.01.

FIGURE S4 Diagram outlining the potential mechanism of BM-MSCs enhancing astrocytes antioxidative function via the Cx43/Nrf2/HO1 axis. BM-MSCs induced Cx43 upregulation, PKCα phosphorylation, Nrf2 stabilization and nuclear translocation, and upregulation of HO-1, then restraining ROS accumulation and cell apoptosis.
