# Supplementary figures and images for: BM-MSC Transplantation Alleviates Intracerebral Hemorrhage-Induced Brain Injury, Promotes Astrocytes Vimentin Expression, and Enhances Astrocytes Antioxidation via the Cx43/Nrf2/HO-1 Axis
Source: Front Cell Dev Biol. 2020 May 8;8:302. doi: 10.3389/fcell.2020.00302 (PMC7227447; doi:10.3389/fcell.2020.00302)

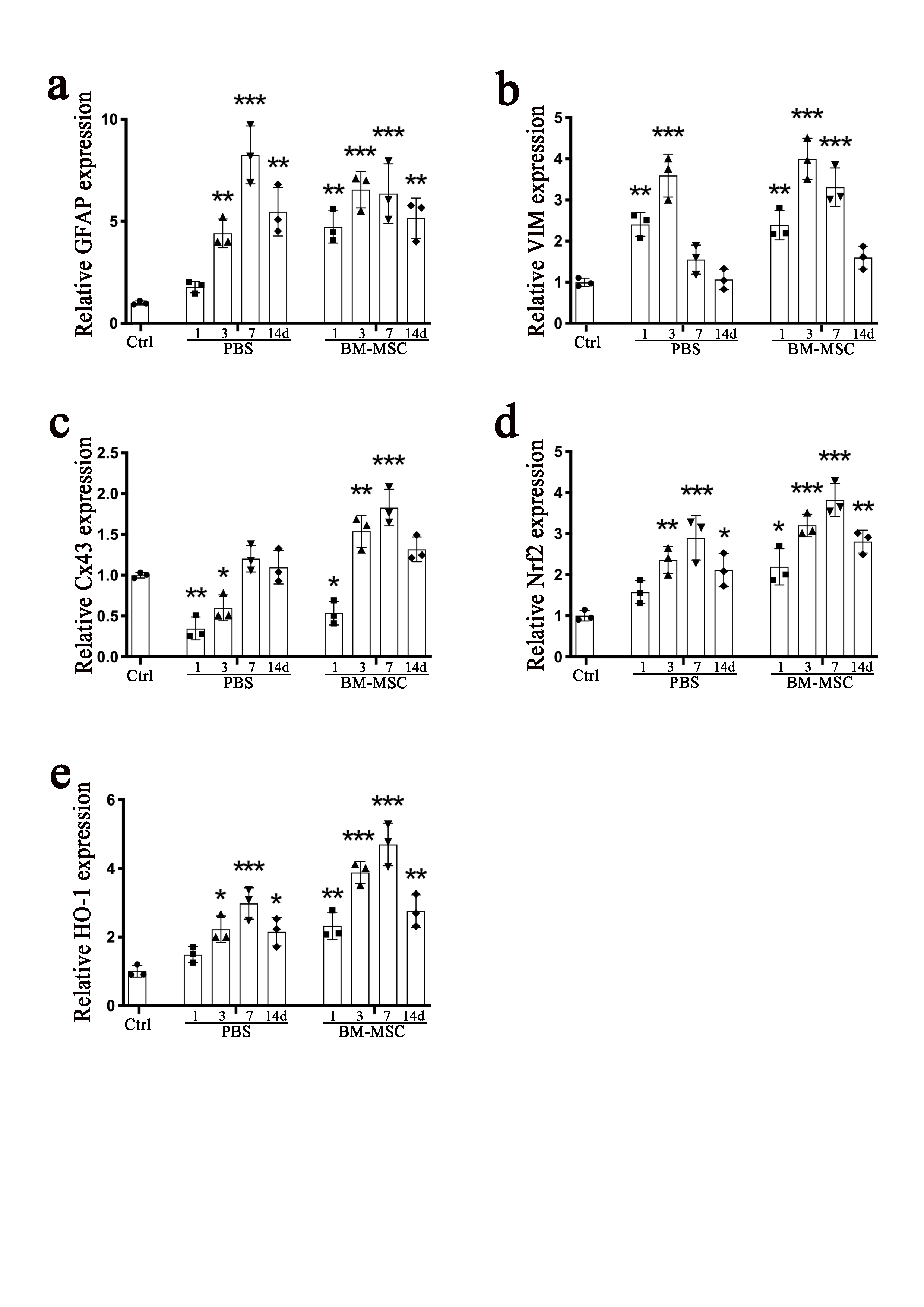

Supplement: FIGURE S1 — Western blotting analysis of Cx43, GFAP, VIM, Nrf2 and HO-1 expression in ICH mouse brain of control, PBS, and BM-MSCs treatment at 1, 3, 7, and 14 days. (A–E) The results of densitometric analysis of the bands. All data are displayed as means ± SD (n = 3). The difference between groups was analyzed using One-way ANOVA test. *p < 0.05, **p < 0.01, ***p < 0.001, compared with control. [file Image_1.JPEG]

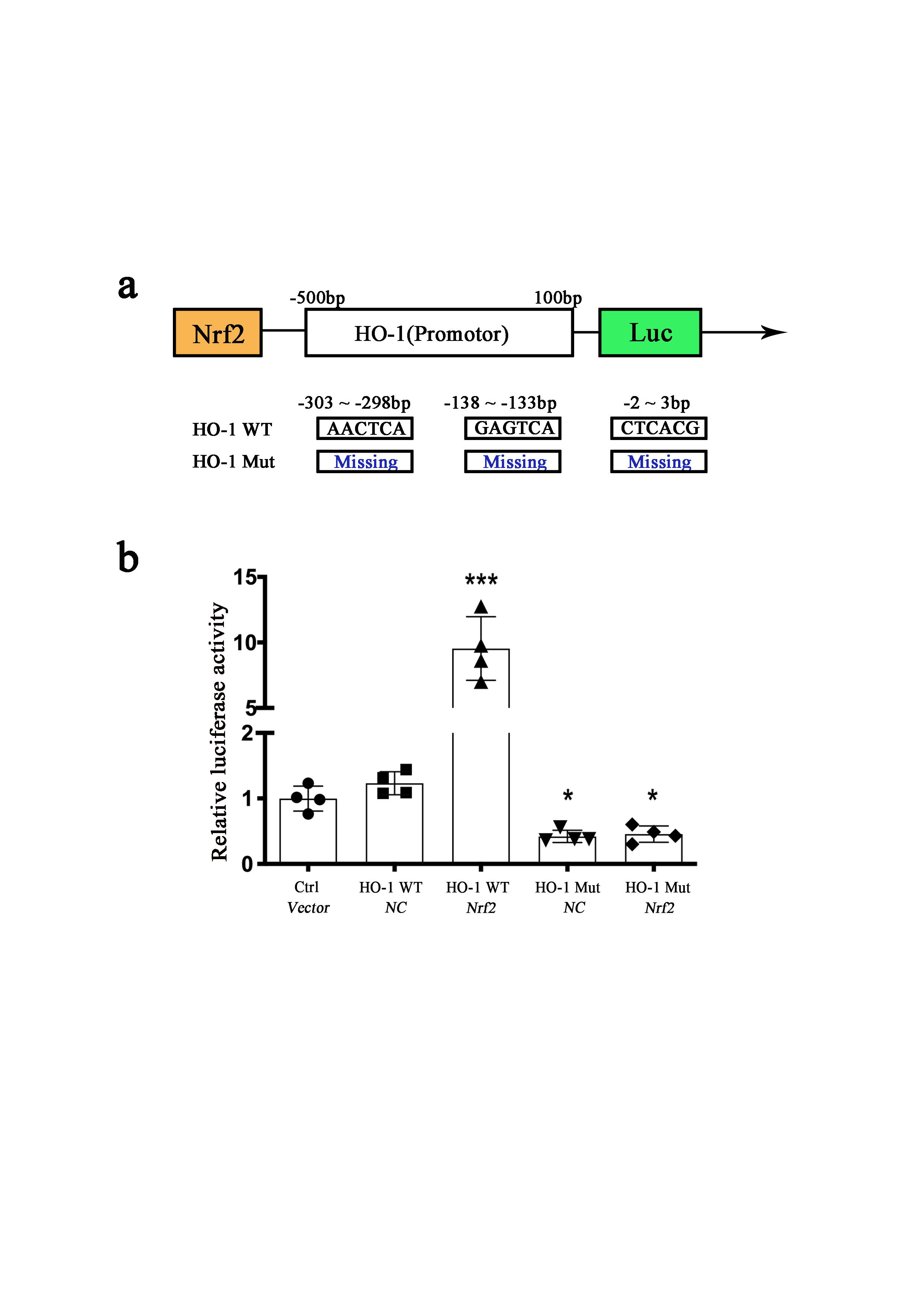

Supplement: FIGURE S2 — The diagram and results of luciferase activity analysis between Nrf2 and HO-1 in primary astrocytes. (a) The HO-1 wildtype group (WT) and the HO-1 mutation group (Mut) were set up to verify our experiments. Three mutation sites were set at – 303 ∼ – 298 bp, – 138 ∼ – 133 bp, and – 2 ∼3 bp, respectively (from −500 bp upstream to 100 bp downstream). (b) All data are displayed as means ± SD (n = 4). The difference between groups was analyzed using One-way ANOVA test. *p < 0.05, ***p < 0.001, compared with control. [file Image_2.JPEG]

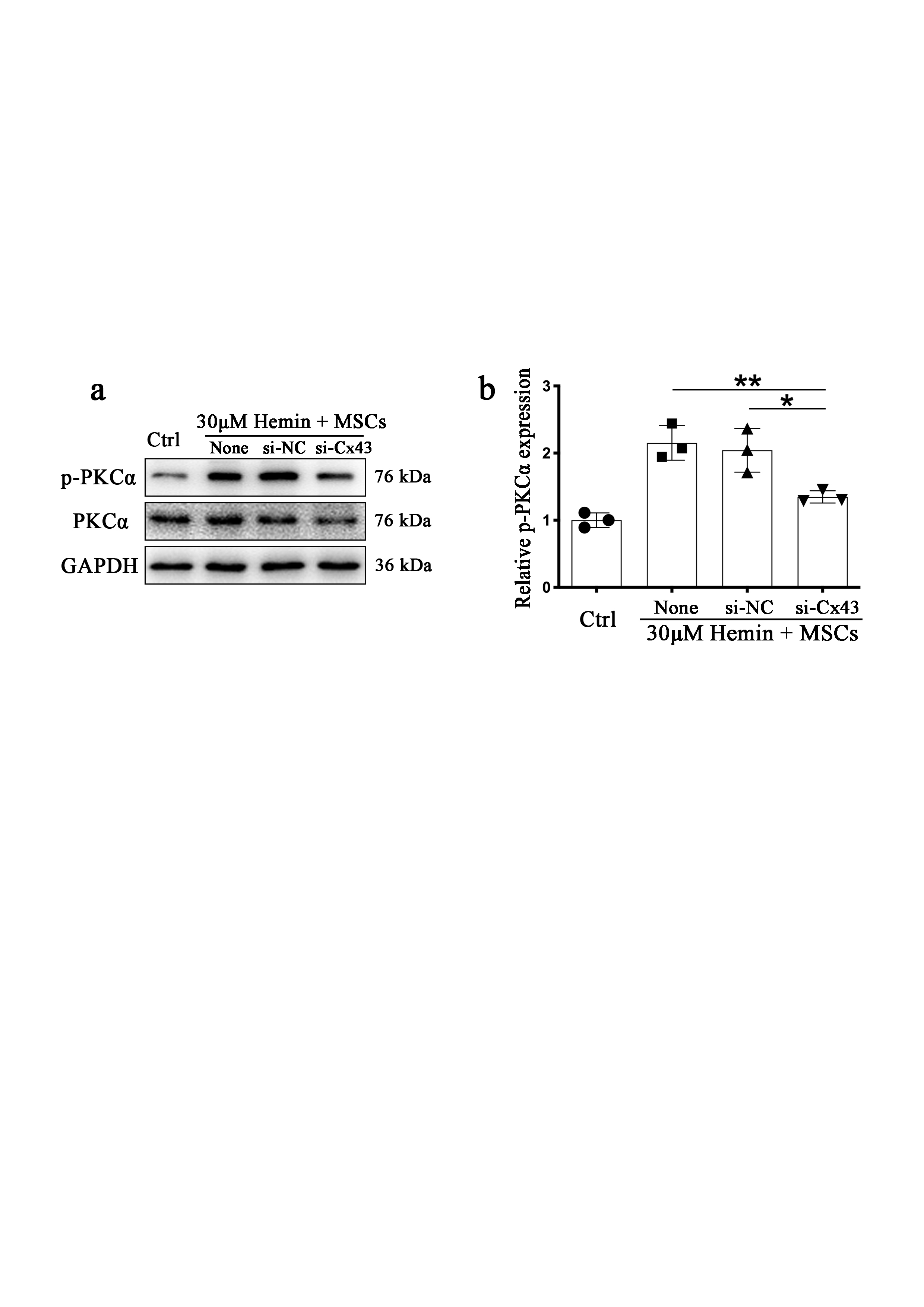

Supplement: FIGURE S3 — Cx43 knockdown suppressed BM-MSCs-induced p-PKCα expression. (a,b) Western blotting analysis of p-PKCα and PKCα expression in control, si-NC, si-Nrf2 transfected astrocytes. All data are displayed as means ± SD (n = 3). The difference between groups was analyzed using One-way ANOVA test. *p < 0.05, **p < 0.01. [file Image_3.JPEG]

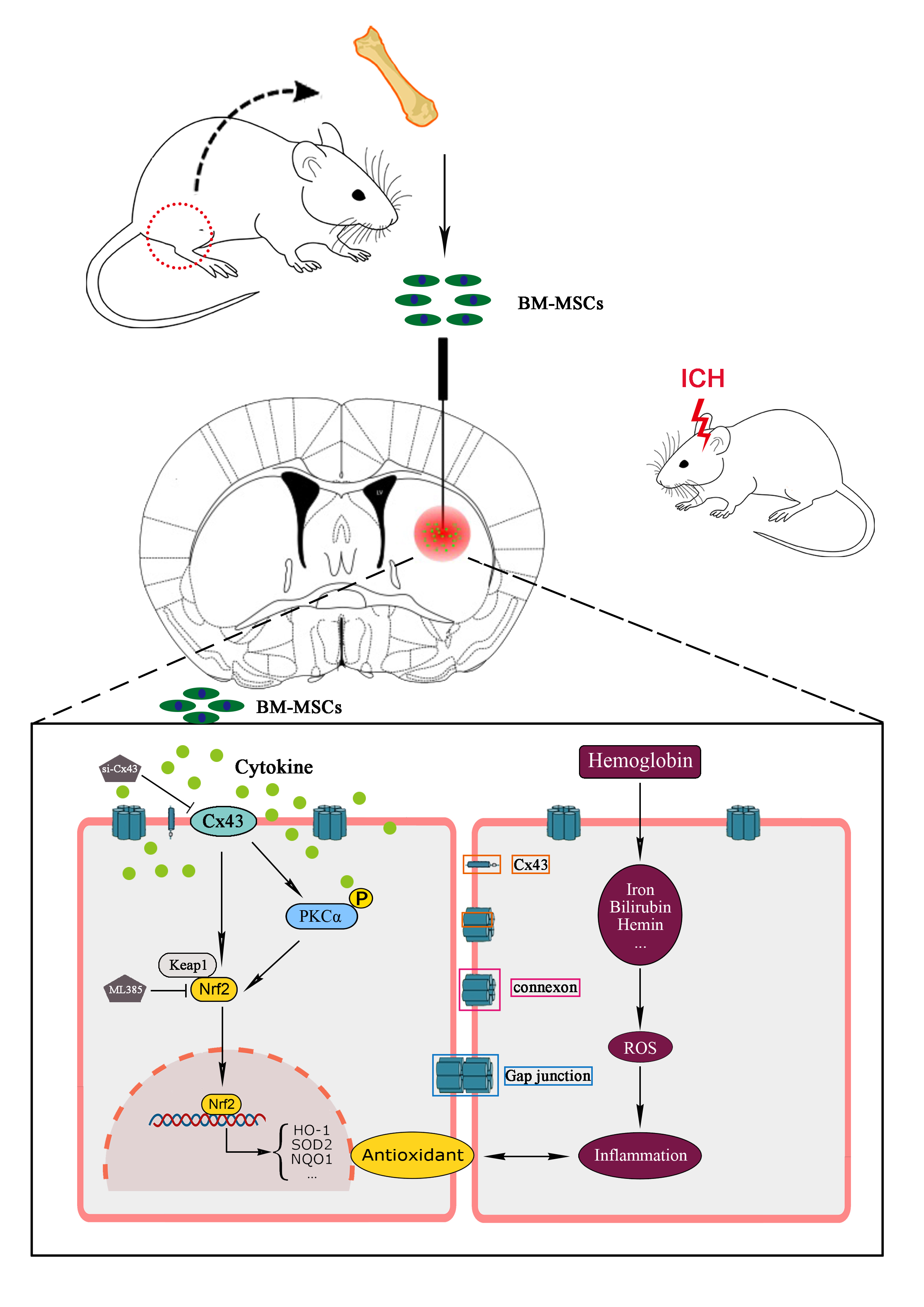

Supplement: FIGURE S4 — Diagram outlining the potential mechanism of BM-MSCs enhancing astrocytes antioxidative function via the Cx43/Nrf2/HO1 axis. BM-MSCs induced Cx43 upregulation, PKCα phosphorylation, Nrf2 stabilization and nuclear translocation, and upregulation of HO-1, then restraining ROS accumulation and cell apoptosis. [file Image_4.JPEG]
